# Identification and Management of Persistent Stimulation-Induced Dyskinesia Associated with STN DBS: The See-Saw Dilemma

**DOI:** 10.5334/tohm.780

**Published:** 2023-08-28

**Authors:** Matthew A. Remz, Joshua K. Wong, Justin D. Hilliard, Tracy Tholanikunnel, Ashley E. Rawls, Michael S. Okun

**Affiliations:** 1 Fixel Institute for Neurological Diseases Campus, University of Florida, Gainesville, FL, USA; 2 Department of Neurology, University of Florida, Gainesville, FL, USA; 3 Department of Neurosurgery, University of Florida, Gainesville, FL, USA

**Keywords:** DBS, STN, dyskinesia, stimulation-induced dyskinesia, Parkinson’s disease

## Abstract

**Clinical vignette:**

A 73-year-old woman with Parkinson’s disease (PD) underwent implantation of bilateral subthalamic nucleus deep brain stimulators (STN-DBS) to address bilateral upper extremity medication-refractory tremor. Post-operatively, she experienced a “see-saw effect” where small increases in stimulation resulted in improvement in one symptom (tremor) with concurrent worsening in another (dyskinesia).

**Clinical dilemma:**

SID is usually considered a positive predictor of DBS outcome. However, there are cases where SID cannot be optimized. Lead location and pre-operative characteristics may contribute to this adverse effect. If the combination of programming and medication adjustments fails to resolve SID, what can be done to “rescue” the outcome?

**Clinical solution:**

Management of SID requires a gradual and steadfast programming approach. Post-operative lead localization can guide advanced programming and decision-making. Rescue surgical interventions may be considered.

**Gap in knowledge:**

In cases where SID is persistent despite deploying persistent optimization strategies, there is limited guidance on next steps.

## Clinical vignette

A 73-year-old woman with a 23-year history of tremor-predominant PD presented for evaluation of refractory tremors which co-occurred with medication-induced dyskinesia despite exhaustive attempts at adjusting medication types, timing and dose. She underwent bilateral subthalamic nucleus (STN) deep brain stimulation (DBS) at the University of Florida Fixel Institute for Neurological Diseases Campus. The STN target was selected on the basis of interdisciplinary review and in conjunction with the patient’s priority of maximal and immediate tremor control. Her initial post-operative monopolar programming threshold review (Table 1) revealed significant stimulation-induced dyskinesia (SID) which occurred at low current density in both of her STN DBS leads. The SID could be induced by three of the four contacts on her left DBS lead and at the best therapeutic contact on her right STN DBS lead. Local field potential data acquired while increasing delivered current demonstrated an increase in oscillations in the gamma range, which correlated with dyskinesia (Figure 3). Following 6 months of comprehensive programming trials, SID remained persistent and bothersome, albeit moderately improved. Despite optimization, any slight adjustments to her carbidopa/levodopa or to her DBS settings exacerbated the hyperkinesia. Increases in current density during programming were necessary to capture upper extremity tremor, however these changes consistently triggered bilateral lower extremity dyskinesia. Amantadine and trihexyphenidyl, both of which were part of her regimen prior to surgery, were discontinued by the patient due to the emergence of a subjective generalized ill feeling which resolved following medication discontinuation.

**Table 1:** Monopolar Threshold Programming Review at the Initial DBS Programming Session.


CONTACT NUMBER	LEFT STN DBSTHRESHOLD (IN MA)	SIDE EFFECT	RIGHT STN DBSTHRESHOLD (IN MA)	SIDE EFFECT

0	1.0	Light-headedness, Paresthesias at 1.2 mA	1.8	Paresthesias of the hand

1	0.9	Dyskinesia	2.3	Dyskinesia

2	0.5	Dyskinesia	2.5	Arm tightness, dysarthria

3	1.5	Dyskinesia	2.6	Arm tightness, dysarthria


Note the low current densities necessary to elicit dyskinesia in the initial DBS programming session. Thresholds were obtained at a pulse width of 90 µS and a frequency of 135 Hz.

**Figure 1:**
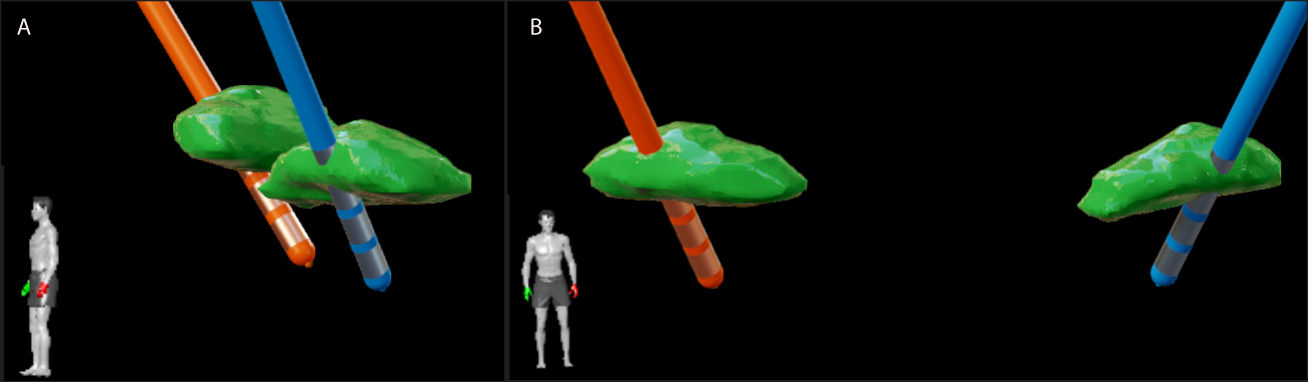
**Lead locations within the STN. (1a)** Top, lateral view of the right STN (orange) and left STN (blue) lead locations within the STN region (green). **(1b)** Bottom, anterior view of the right STN (orange) and left STN (blue) lead locations within the STN region (green). These locations are relatively ventral and anterior within the target region.

Consistently, she reported SID at electrical current levels that were applied below 1 mA. The location of her DBS lead and active contacts were calculated using co-registration of pre-operative volumetric MRI and a post-operative high resolution CT scan (BrainLab Elements, Westchester, IL., USA)) (Figure 1). Lead localization demonstrated relatively anterior and ventral lead locations within the STN region.

## Clinical dilemma

This case demonstrates the potential for STN-DBS to result in persistent SID. Initial post-operative counseling of this patient focused on the usual favorable outcome associated with SID. However, a “see-saw effect” can emerge where small increases in stimulation result in improvement in one symptom (tremor) with concurrent worsening in another (dyskinesia). Alternatively, reduction in medication led to worsening of tremor and improvement in dyskinesia. The clinician may thus apply programming and medication strategies but persistently be unable to balance the clinical see-saw. The question in this case involves the potential impact of underlying disease characteristics, the impact of lead location and the region of stimulation, and weighing the value of continued stimulation that produces SID. This guides shared decision making around consideration of a rescue GPi DBS, lead relocation, or a pallidotomy procedure.

## Clinical solution

Managing SID is usually approached with the clinician employing slow ramping of stimulation amplitude over several months. Many experts minimize pulse width and frequency, focusing on deploying stimulation to more dorsal regions on the DBS lead. Additionally, directional programming, bipolar stimulation, tripolar stimulation, and interleaving may be trialed. The imaging in this case revealed a relatively ventral and anterior location for both DBS leads. The team used the measured lead localization to “steer current” into a potentially more favorable and more dorsal lateral location for potential optimal neuromodulation. In this case, the programming was complex, comprehensive and time consuming.

Ultimately, a combination of double monopolar and directional stimulation with a reduced pulse width and a reduced frequency was employed (Table 2). Frustratingly, while lower extremity tremor was adequately captured with programming, there was a not a DBS setting which could be identified to consistently capture upper extremity tremor without inducing some degree of lower extremity dyskinesia. Thus, the primary goal became a compromise between tremor suppression and some level of SID (below a bothersome or debilitating threshold).

**Table 2:** Optimized DBS Settings.


	CONTACTS	AMPLITUDE (MA)	PULSE WIDTH (µS)	FREQUENCY (HZ)

Left STN: Interleaving, Bipolar	3– C+	1.3	70	150

	3– 2+	0.6	20	60

Right STN Directional, Double Monopolar	11- 9c–	1.4 11– (1.2) 9c– (0.4)	60	150


The post-operative UPDRS part III motor score at the initial programming session, one month following lead placement was 48 off medication and off stimulation. Her 6-month post-bilateral optimized DBS UPDRS part III motor sub-score was 45 off medication/off stimulation and 13 on medication/on stimulation.

A video showing her response to DBS and medications is provided (Video Segment 1) as is a table summarizing the optimized DBS settings (Table 2).

**Video Segment 1:** **Stimulation induced dyskinesia and response to optimized programming.** The patient manifested tremor that was severe when both medication and stimulation were “off”. There was a manifested reduction in tremor, but increased dyskinesia in the medication “on”, stimulation “off” state and in the medication “off”, stimulation “on” state. Following optimization of DBS programming, there was no tremor, but there was mild dyskinesia, in the medication and stimulation “on” state.

Due to her persistent, bothersome SID, she was offered and is considering a staged approach to adding “rescue” GPi leads, beginning with the left side for the more bothersome right hemibody. This could be done either by leaving the current leads in place or removing and replacing them with leads in the GPi target.

## Gaps in Knowledge

### Localization of dyskinesia within the STN

Dyskinesia associated with STN-DBS has been widely reported in PD. The dorsolateral and posterolateral regions of applied stimulation have been postulated to underpin the effect, however there are few cases with details for subjects with persistent SID [1,2]. Notably, there was a single case of persistent SID which resolved when the DBS lead was moved from a more ventral to a more dorsal location [3].

Dyskinesia may occur in other non-PD related hyperkinetic movement disorders. In one recent series of patients with Meige syndrome who were treated with STN-DBS, SID was associated with more ventral (including substantia nigra pars reticulata) rather than the dorsal regions usually observed in SID PD cases. Though the study methods were suboptimal in the application of STN segmentation and relied on stereotactic coordinates, they do raise the important question of influence of the underlying disorder on the SID [4]. Supportive of this notion is an example of STN DBS in obsessive compulsive disorder (OCD) which revealed a more anterior location of neuromodulation within STN, which was observed to trigger hyperkinesia [5,6]. Finally, STN related SID was the most common “limiting side effect” in a prospective study of isolated dystonia DBS. The cases were likely associated with more dorsolateral neuromodulation than ventral locations [7,8]. Therefore, we must ask the question whether SID associated with STN-DBS leading to persistent hyperkinesia is a distinct entity from the classical transient post STN-DBS SID, which is usually localized to the dorsolateral sensorimotor STN. Tractography, connectomics, and a closer examination of the neural networks may provide clues to answer this question, however individual baseline disease characteristics may also be a critically important consideration.

### How to manage persistent stimulation induced STN related dyskinesia

Perhaps the best chance to avoid SID may be the recognition of potential pre-operative risk factors, especially “brittle dyskinesia” (hyperkinesia resulting from the use of ≤ one 25/100 mg tablet of carbidopa/levodopa per dose). Once persistent SID emerges, using empirical trials of dorsal stimulation, bipolar, tripolar or directional stimulation all may provide potential management solutions [9,10]. Ramping stimulation slowly over many months is considered a mainstay of therapy and in some cases the SID effect may habituate over time, facilitating the use of larger current densities. Dorsal stimulation has been proposed to reduce SID, perhaps through targeting of pallidofugal fibers [11]. However, in many cases the leads have already been placed in the dorsolateral position, and in other cases the trajectory may not include adequate access to the pallidofugal fiber pathways.

Many experts may be swayed to use STN DBS if tremor suppression is the primary goal. However, a recent meta-analysis of STN and GPi DBS has revealed similar tremor suppression outcomes and thus it may be more important to consider “brittleness” and how disabling dyskinesia is pre-operatively [12,13]. There are a few reports of benefit in cases of SID with dorsal relocation of STN leads and the use of GPi rescue leads [3,12,14]. There are no reports of pallidotomy.

### Expert commentary

**Figure 2:**
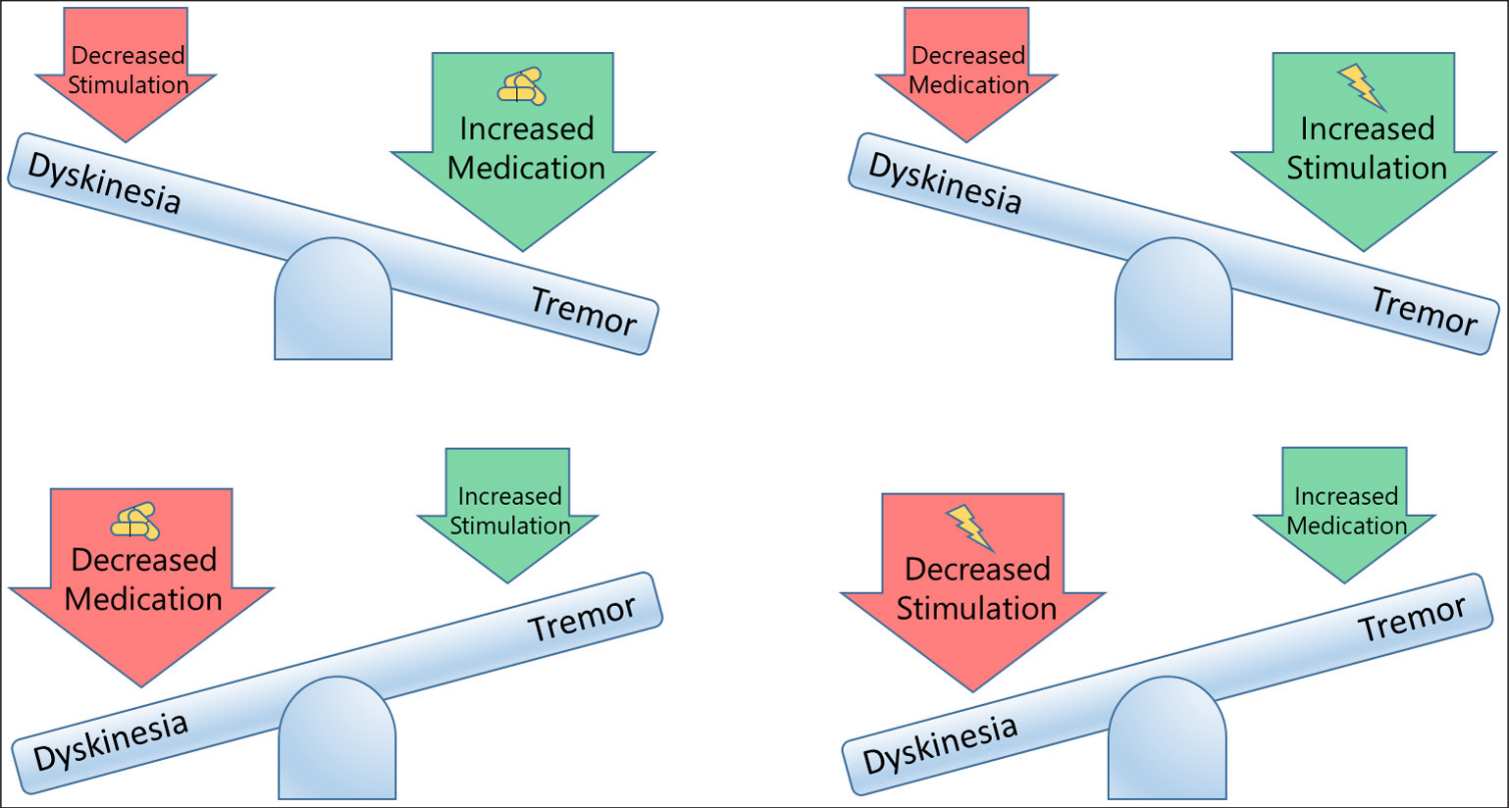
**The See-Saw Dilemma in STN Related Stimulation Induced Dyskinesia.** In cases of STN induced SID, the clinician is challenged to balance the see-saw. Small increases in stimulation result in improvement in one symptom (tremor) with worsening in another (dyskinesia). Alternatively, reduction in medication, may lead to worsening of tremor and improvement in dyskinesia. The clinician may apply programming and medication strategies to attempt to balance the see-saw.

This case underscores the importance of recognizing persistent SID and the “see-saw dilemma” which may emerge during management. There is a paucity of published studies to guide DBS programming and to advise concurrent medication adjustments. The see-saw effect (Figure 2) refers to the frustrating attempts by a clinician to achieve therapeutic equilibrium. The presence of pre-operative “brittle dyskinesia,” as in this case, may have been a missed opportunity to pursue a GPi target.

**Figure 3:**
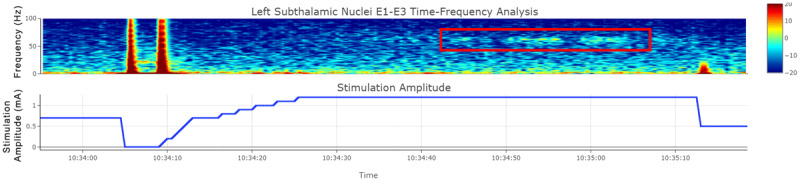
**Gamma Band Increase from STN DBS: Time-Frequency Spectrogram of Local Field Potential Data from the Left STN lead.** The application of stimulation using 1.5 mA of current applied to the left STN DBS lead (bottom tracing, blue line) induced visualized dyskinesia on physical examination that correlated with the onset of a band of increased gamma frequency (30–100 Hz) activity (red box in the top tracing).

GPi is a well-known dyskinesia suppressor. Well-placed leads in the GPi target afford the managing clinician greater latitude in the administration of higher doses of levodopa, while simultaneously “ramping up” the total amount of stimulation delivered. The clinicians in this case did their best to manage persistent STN SID through slow ramping of stimulation over time, dorsal stimulation, bipolar stimulation, tripolar stimulation and attempts at applying a directional DBS strategy. In our experience, some cases of SID will resolve with the application of slow ramping (e.g. 0.1–0.2 mA per week).

Even when employing these strategies, there are instances where STN lead revision or the addition of a GPi rescue lead will be necessary. In the case of suboptimal lead location, the risks and benefits of repositioning the STN lead into a more dorsolateral position should be weighed. In some circumstances, STN stimulation may be producing partial benefit below the threshold for SID. In this circumstance, maintaining the contribution that STN stimulation provides, while adding a GPi lead to produce concurrent stimulation may aid in a patient’s overall therapy and offer more long term programming options. In most of these circumstances, when there is concern for losing positive STN benefit, maintaining the STN lead and adding a GPi lead, rather than revising the STN lead alone, is the safer and higher-yield approach to address persistent SID.

In this case, the anterior and ventral location of the STN DBS leads could argue that repositioning dorsolateral may resolve the issue. Data supporting direct dyskinesia suppression with GPi stimulation may reduce clinical equipoise for a return to the STN target. Finally, this case adds a measure of circumstantial support to the argument that a baseline propensity for brittle dyskinesia should be considered when choosing a brain target (STN vs. GPi) during an interdisciplinary pre-operative DBS workup.
